# Efficacy of Multimodal Pain Control Protocol in the Setting of Total Hip Arthroplasty

**DOI:** 10.4055/cios.2009.1.3.155

**Published:** 2009-08-17

**Authors:** Kyung-Jae Lee, Byung-Woo Min, Ki-Cheor Bae, Chul-Hyun Cho, Doo-Hyun Kwon

**Affiliations:** Department of Orthopedic Surgery, School of Medicine, Keimyung University, Daegu, Korea.

**Keywords:** Total hip arthroplasty, Multimodal pain control protocol, Visual analogue scale

## Abstract

**Background:**

This study evaluated the benefits and safety of a multimodal pain control protocol, which included a periarticular injection of local anesthetics, in patients undergoing total hip arthroplasty.

**Methods:**

Between March 2006 and March 2007, 60 patients undergoing unilateral total hip arthroplasty were randomized to undergo either a multimodal pain control protocol or a conventional pain control protocol. The following parameters were compared: the preoperative and postoperative visual analogue scales (VAS), hospital stay, operative time, postoperative rehabilitation, additional painkiller consumption, and complication rates.

**Results:**

There was no difference between the groups in terms of diagnosis, age, gender, and BMI. Although both groups had similar VAS scores in the preoperative period and on the fifth postoperative day, there was a significant difference between the groups over the four-day period after surgery. There were no differences in the hospital stay, operative time, additional painkiller consumption, or complication rate between the groups. The average time for comfortable crutch ambulation was 2.8 days in the multimodal pain control protocol group and 5.3 days in the control group.

**Conclusions:**

The multimodal pain control protocol can significantly reduce the level of postoperative pain and improve patients' satisfaction, with no apparent risks, after total hip arthroplasty.

Total hip arthroplasty (THA) is performed for pain relief, normal joint motion, and a deformity correction of the hip. However, its efficacy is often limited by perioperative pain management.^1^-5) In some studies, proper pain control is not obtained in more than half of patients, who undergo THA.^6^,7) Failure of postoperative pain management directly affects rehabilitation of patients. Many authors have suggested a variety of solutions for postoperative pain and demonstrated their remarkable efficacy, but the complications associated with the overuse of narcotics^8^,9) and nerve blocks cannot be avoided.^3^,^10^-13) Therefore, in an attempt to provide effective pain management and reduce these drug-related complications, we developed a multimodal pain control protocol using a mixture of drugs with different mechanisms of action in patients undergoing THA and examined its efficacy.

## METHODS

Of the patients who underwent primary THA performed in our hospital between March 2006 and March 2007, 60 patients were enrolled in this study. The exclusion criteria included those with severe psychiatric disorders, a history of drug addiction, allergy to any study medication, cerebral infarction or a severe neurological lesion, and severe heart or renal disease. The study population was divided randomly into 2 groups: Group I (n=30) received our multimodal pain management including periarticular injections of multimodal drugs; and group II received only standard pain management. The results of the two protocols were compared prospectively in terms of the level of pain measured before and until 5 days after surgery, hospital stay, operative time, postoperative rehabilitation, postoperative consumption of parenteral pain medication, and complications of pain medications, such as nausea (Table 1).

The mean age of the patients was 53.1 years (range, 31 to 76 years). There were 36 males and 24 females. The presumed cause was osteonecrosis of the femoral head in 43 cases, acetabular dysplasia with advanced osteoarthritis in 8 cases, sequelae of Legg-Calvé-Perthes' disease in 5 cases, and sequelae of pyogenic arthritis in 4 cases. There were no differences in gender, age, presumed cause, height, weight, and BMI between the two groups (Table 2).

Preemptive analgesia was performed for central sensitization 2 hours before surgery in group I while it was not applied in group II. In all cases, surgery was performed under general anesthesia. In group I, epidural anesthesia and a periarticular injection of multimodal drugs were also performed intraoperatively. In group II, intravenous patient-controlled analgesia was used postoperatively. Isoflurane was used for inhalational anesthesia. For intravenous patient-controlled analgesia, different doses of morphine were administered depending on the patient's age, height, and weight. All procedures were carried out by the same surgeon using a minimally invasive anterolateral approach according to a standard protocol. The periarticular injection (90 ml) contained 5 mg of morphine HCl, 40 mg of methylprednisolone, and 6.8 mg of ropivacaine dissolved in 70 ml of 0.9% normal saline. One-third (30 ml) of the mixture was injected before inserting the prosthesis, another third (30 ml) was administered immediately before reducing the prosthesis into the synovial sheath, joint capsule, deep fascia, and damaged muscle layer, and the remainder (30 ml) was infused into the subcutaneous tissues and adipose layer (Table 3). For postoperative pain management, all patients were given regular oral nonsteroidal anti-inflammatory drugs (NSAIDs) and parenteral analgesics were used when needed. In group I (multimodal pain control group), more than two analgesics with different mechanisms of action were administered orally for postoperative pain control until 2 days after surgery (Table 4).

The pain was assessed by the patients using a 100 mm visual analogue scale (VAS; 0 mm = no pain, 100 mm = unbearable pain). They were asked to make measurements at a specific time before surgery, immediately after sur gery, and from the 1st to the 5th postoperative day. Depending on the level of pain, the patients were assigned to different postoperative rehabilitation programs ranging from quadriceps strengthening to straight leg raising. The days on which comfortable ambulation was achieved with the aid of crutches were also recorded. The doses of additional parenteral analgesics prescribed on an asneeded basis were assessed until the 5th postoperative day. The occurrence of complications such as nausea, vomiting, and surgical site infection was also investigated. Statistical analysis was performed suing a Student's t-test and Chi-square test. Statistical significance was defined as *p* < 0.05.

## RESULTS

### Visual Analogue Scale

**Fig. 1:**
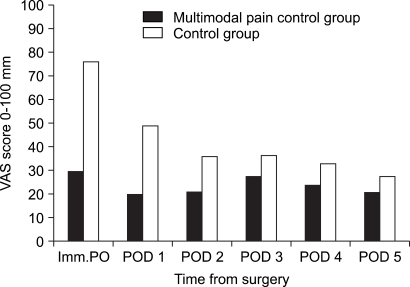
Summary of the visual analogue scale (VAS) for pain. Imm. PO: Immediate postoperatively, POD1, POD2, POD3, POD4, and POD5: one, two, three, four, and five days postoperatively.

The average VAS score in groups I and II was 2.3 and 2.6 preoperatively, indicating no significant difference between the two groups (*p* = 0.263). The mean VAS scores measured every day at a specific time from immediately after surgery until the 4th postoperative day were respectively, 3.0, 2.0, 2.1, 2.7, and 2.4 in group I and 7.6, 4.9, 3.6, 3.6, and 3.3 in group II. Significant pain relief was noted in group I (*p* < 0.05). The mean VAS score obtained on the 5th postoperative day was slightly lower in group I than in group II (2.1 and 2.7, respectively), but there was no significant difference between the two groups (*p* = 0.275) (Fig. 1).

### Hospital stay and Operative Time

The mean hospital stay was 18.8 ± 6.0 days in group I using our multimodal pain control protocol and 20.6 ± 6.3 days in group II. There was no significant difference between the two groups (*p* = 0.190). The mean operative time from the skin incision to closure was 114.5 minutes (range, 60 to 150 minutes) in group I with periarticular injections and 110.1 minutes (range, 60 to 150 minutes) in group II. This shows that the periarticular injections extended the surgery time slightly but the difference is not statistically significant (*p* = 0.442).

### Postoperative Rehabilitation

The average time required for the patients to be able to perform straight leg raising excercises without pain and comfortable ambulation with the aid of crutches was 2.8 days and 5.3 days in groups I and II, respectively. This indicates that, group I with our multimodal pain control protocol showed more rapid recovery than group II (*p* = 0.000).

### Additional Consumption of Analgesics and Complication Rate

The two groups consumed a similar level of additional analgesics as a supplement to the regular intake of NSAIDs prescribed until the 5th postoperative day, 2.5 and 3.1 times in group I and II, respectively; (*p* = 0.236). There were 9 and 12 medication-related complications, such as nausea and vomiting, in groups I and II, respectively, showing no significant difference (*p* = 0.725). There were no cases of wound infection or a delay in wound healing due to the extended period of drainage.

## DISCUSSION

As a reflection of the growing interest in pain, the American Pain Society designated pain as the 5th vital sign and stated that the success or failure of an operation is determined by postoperative pain management. Pain is a unique experience for each individual and the responses to postoperative pain management vary. Many authors developed variety of methods, such as continuous epidural anesthesia, injection of analgesics into the joint cavity, intravenous patient-controlled analgesia, and nerve blocks^12^,^14^-16) and described their efficacy in postoperative pain management. Some authors reported that a single pain management method had only limited effects.^1^-5) In addition, various complications related to medications, such as nausea, vomiting, respiratory failure, hypotension, paralysis and dysuria, were also reported.^10^-^13^,^17^-19)

Pain is a sensation perceived through several steps after tissue trauma: transduction, transmission, modulation in the spinal cord, and perception in the cerebral cortex. Multimodal pain control protocols using a combination of analgesic regimens with different mechanisms of action have been designed to promote effective pain control and reduce medication-related side effects.^16^,20) The protocols also include preoperative patient education, proper anesthesia, and minimally invasive surgery for the least tissue damage. There has been increasing interest in multimodal pain control protocols and several studies have reported satisfying results.^20^-23) In this study, the multimodal pain control protocol also reduced significantly the level of pain from immediately after surgery to the 4th postoperative day, particularly to the 2nd postoperative day. Unfortunately, the VAS score was approximately 3 (bearable pain) since the 3rd postoperative day, which is statistically but not clinically significant. This is believed to be the reason why there was no difference in the dose of postoperative parenteral analgesia between the two groups.

Our multimodal pain control protocol can be characterized by the use of preemptive analgesia, epidural anesthesia and a periarticular injection during surgery, and various medications after surgery. Reports have shown that preemptive analgesia prevents central sensitization and reduces the level of postoperative pain.^24^-26) Parvataneni et al.27) reported that a periarticular injection of multimodal drugs is one of the most important procedures in multimodal pain control protocol. According to Vendittoli et al.,28) an injection into injured or stretched nerves or tissues under the guidance of a surgeon can lead to a direct block of the nerves. The postoperative pain control procedures using variety of pain medications is also an inevitable element in maintaining the efficacy of pre- and intraoperative pain control procedures. The major medications used in this study included oxycodone CR, COX-2 selective inhibitor, acetaminophen, morphine HCl, methylprednisolone (Depo-Medrol), and ropivacaine. Among them, Oxycodone CR was used to activate the mu-opioid receptors to inhibit the release of pain transmitters. The COX-2 selective inhibitor was used as a basic pain medication and an anti-inflammatory agent inhibiting isoenzyme and prostaglandin production. Morphine HCl, methylprednisolone, and ropivacaine, which were components of the periarticular injection cocktail, activated directly the mu-opioid receptor near the surgical site inhibiting the local inflammatory response and relieving the pain by preventing the production of pain transmitters. The concomitant use of these drugs resulted in effective pain management by disrupting the various pathways of pain.

However, the use of epidural anesthesia, which is an invasive procedure, and the dditional expenses should be taken into consideration when applying a multimodal pain control protocol. In addition, although there were no complications associated with periarticular injections in this study, Vendittoli et al.28) warned of the risk of the plasma concentration of local anesthetics, which they observed in some of their study population.

According to Burns et al.,29) various factors such as age, gender, cultural background, education, and expectation of surgery affect the awareness of pain after surgery. Vendittoli et al.28) reported that the use of a multimodal pain control protocol contributed reduction in the use of narcotics. Unfortunately, due to the small study population, this study could not examine the association between these factors. More studies on the relationship between pain control protocols and narcotics in Koreans will be needed to increase the efficacy of pain management in Korea.

In conclusion, multimodal pain control protocols using a mixture of more than two drugs with different mechanisms of actions effectively improve the patients' satisfaction by providing early postoperative pain relief without increasing the risk of complications and prolonging the surgery time in patients undergoing THA.

## Figures and Tables

**Table 1:**
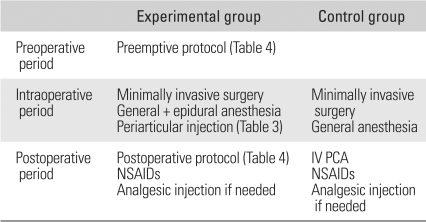
Schema of This Study

IV PCA: Intravenous patient-controlled analgesia, NSAIDs: Nonsteroidal anti-inflammatory drugs.

**Table 2:**
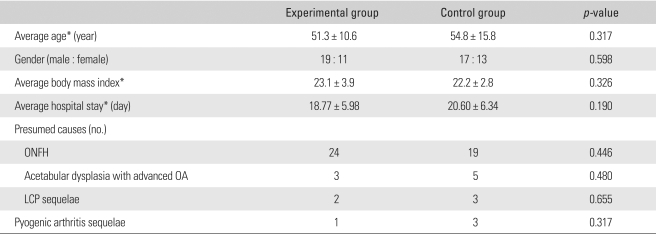
Demographic Data

^*^All values are presented as the mean ± standard deviation. ONFH: Osteonecrosis of femoral head, OA: Osteoarthritis, LCP: Legg-Calvé-Perthes disease.

**Table 3:**
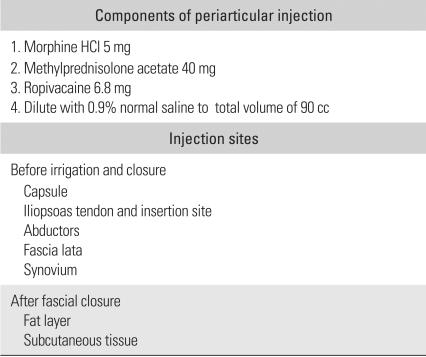
Components and Injection Sites of Intraoperative Periarticular Injection in the Experimental Group

**Table 4:**
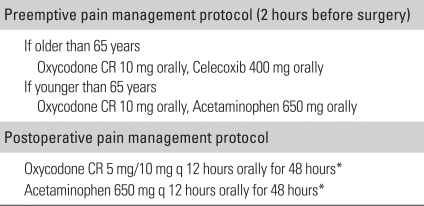
Components and Protocol for Preemptive and Postoperative Pain Control in the Experimental Group

^*^Use alternately.
